# Facets of ICP-MS and their potential in the medical sciences—Part 1: fundamentals, stand-alone and hyphenated techniques

**DOI:** 10.1007/s00216-022-04259-1

**Published:** 2022-08-27

**Authors:** David Clases, Raquel Gonzalez de Vega

**Affiliations:** 1 Nano Mirco LAB, Institute of Chemistry, University of Graz, Graz, Austria; 2 TESLA-Analytical Chemistry, Institute of Chemistry, University of Graz, Graz, Austria

**Keywords:** Inductively coupled plasma–mass spectrometry, Biomonitoring, Elemental bioimaging, Elemental speciation analysis, Isotope ratios, LA-ICP-MS, LC-ICP-MS

## Abstract

Since its inception in the early 80s, inductively coupled plasma–mass spectrometry has developed to the method of choice for the analysis of elements in complex biological systems. High sensitivity paired with isotopic selectivity and a vast dynamic range endorsed ICP-MS for the inquiry of metals in the context of biomedical questions. In a stand-alone configuration, it has optimal qualities for the biomonitoring of major, trace and toxicologically relevant elements and may further be employed for the characterisation of disrupted metabolic pathways in the context of diverse pathologies. The on-line coupling to laser ablation (LA) and chromatography expanded the scope and application range of ICP-MS and set benchmarks for accurate and quantitative speciation analysis and element bioimaging. Furthermore, isotopic analysis provided new avenues to reveal an altered metabolism, for the application of tracers and for calibration approaches. In the last two decades, the scope of ICP-MS was further expanded and inspired by the introduction of new instrumentation and methodologies including novel and improved hardware as well as immunochemical methods. These additions caused a paradigm shift for the biomedical application of ICP-MS and its impact in the medical sciences and enabled the analysis of individual cells, their microenvironment, nanomaterials considered for medical applications, analysis of biomolecules and the design of novel bioassays. These new facets are gradually recognised in the medical communities and several clinical trials are underway. Altogether, ICP-MS emerged as an extremely versatile technique with a vast potential to provide novel insights and complementary perspectives and to push the limits in the medical disciplines. This review will introduce the different facets of ICP-MS and will be divided into two parts. The first part will cover instrumental basics, technological advances, and fundamental considerations as well as traditional and current applications of ICP-MS and its hyphenated techniques in the context of biomonitoring, bioimaging and elemental speciation. The second part will build on this fundament and describe more recent directions with an emphasis on nanomedicine, immunochemistry, mass cytometry and novel bioassays.

**Graphical abstract:**

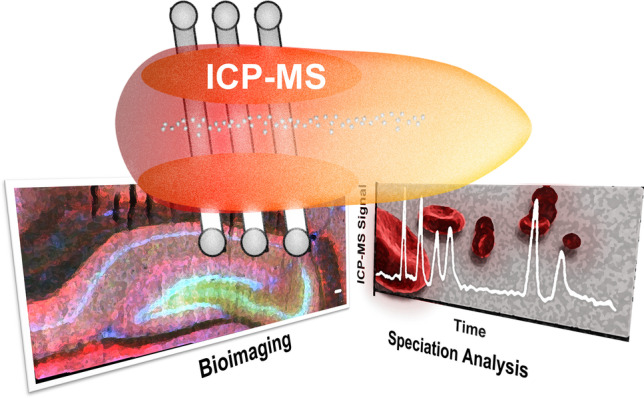

## Introduction

Essential trace elements play a fundamental role in biology and are regulated in a narrow homeostasis. This is relevant for various medical questions where the levels, speciation and spatiotemporal distribution of elements are aberrant and associated with specific pathologies. Targeting and calibrating elements as bio-indicative entities consequently provides opportunities for diagnostics and further promotes a better general understanding of the underlying biochemistry.

Atomic absorption spectroscopy (AAS) developed in the 1950s by Alan Walsh can be seen as the inception of trace element analysis and was absorbed by the medical sciences providing important new pathological and physiological insights. While AAS, among other techniques, is still a prevalent element analytical technique in the clinical landscape, various new techniques have been brought forward over the last decades and promise entirely new perspectives to study the levels of trace elements and their biological impact. Especially, the commercial introduction of inductively coupled plasma–mass spectrometry (ICP-MS) in the early 1980s was a turning point for trace element analysis. ICP-MS does not only outperform most techniques regarding sensitivity, selectivity and dynamic calibration range but further enables elemental speciation analysis and bioimaging when paired with chromatography or laser ablation. The gradual improvement of hard- and software as well as tailored methodologies for ICP-MS enhanced capabilities and figures of merit substantially and provided intriguing avenues to interrogate concentrations of trace elements, their localisation in highly compartmentalised biological structures and their species distributions. Despite the tremendous potential of ICP-MS and its associated techniques and protocols, it is still underutilised in both medical research and routine. One reason for this is certainly the requirement for extensive expertise and personnel skills; however, another reason relates to the fact that new paradigms with impact in the medical sciences were stimulated by technological advances and new methodologies which were brought forward recently within a relatively short time frame.

The scope of ICP-MS was vastly expanded by emerging technologies and methodologies, which resulted in different facets with distinct utility for research and clinical translation as well as potential for new directions in therapy, diagnostics and the development of new pharmaceuticals. This review will tackle the spectrum of studies, applications, opportunities and research directions in ICP-MS and will be divided in two parts. The first part will introduce basic concepts relevant to follow current trends and to recognise the potential of ICP-MS in the medical sciences. Important theoretical and practical considerations are highlighted to provide a tutorial perspective on the working principles, pitfalls and limits of ICP-MS techniques. In its easiest (stand-alone) set-up, ICP-MS is an asset for biomonitoring studies and related advantages and applications will be compared against other established techniques. Hyphenated techniques advanced the scope of ICP-MS significantly forming the fundament for speciation analysis and bioimaging. Concepts, considerations and applications of hyphenated ICP-MS with an emphasis on calibration approaches to gain quantitative insight into biomedical processes will be discussed subsequently. Finally, isotope ratio analysis will be discussed regarding opportunities to promote tracer studies and to study isotope fractionation effects to better the understanding of physiology and pathologies.

The second part of this review will advance on the concepts introduced in the first part and will describe more recent facets of ICP-MS. An emphasis will be given to immunochemical methods which inspired the field of mass cytometry. In this context, current directions and future perspectives regarding clinical utility will be discussed briefly. Another focus will be on single-event ICP-MS which enables the characterisation of nanomaterials and promotes the development of novel nano-scaled platforms for drug delivery and novel imaging probes. Approaches to expand methods to the analysis of endogenous elements and therapeutic elements in individual cells will be considered as well. Finally, methods interlacing immunochemistry and single-event ICP-MS will be evaluated in their ability to tailor new bioassays for the detection of protein- and nucleic acid–based biomarkers.

The aim of this review is to showcase the facets of ICP-MS and their capabilities. Basic concepts and principles will be introduced while selecting applications and examples with biomedical implications or clinical relevance. In this framework, technological milestones, new directions and pitfalls as well as the potential and future perspective will be highlighted. The review targets an interdisciplinary audience of (bio)analytical chemists, biologists, nanotechnologists and medical researchers as well as researchers of various other disciplines operating at the fringe of medicine or interested in the capabilities of ICP-MS.

## Fundamentals: instrumentation and hardware

**Fig. 1:**
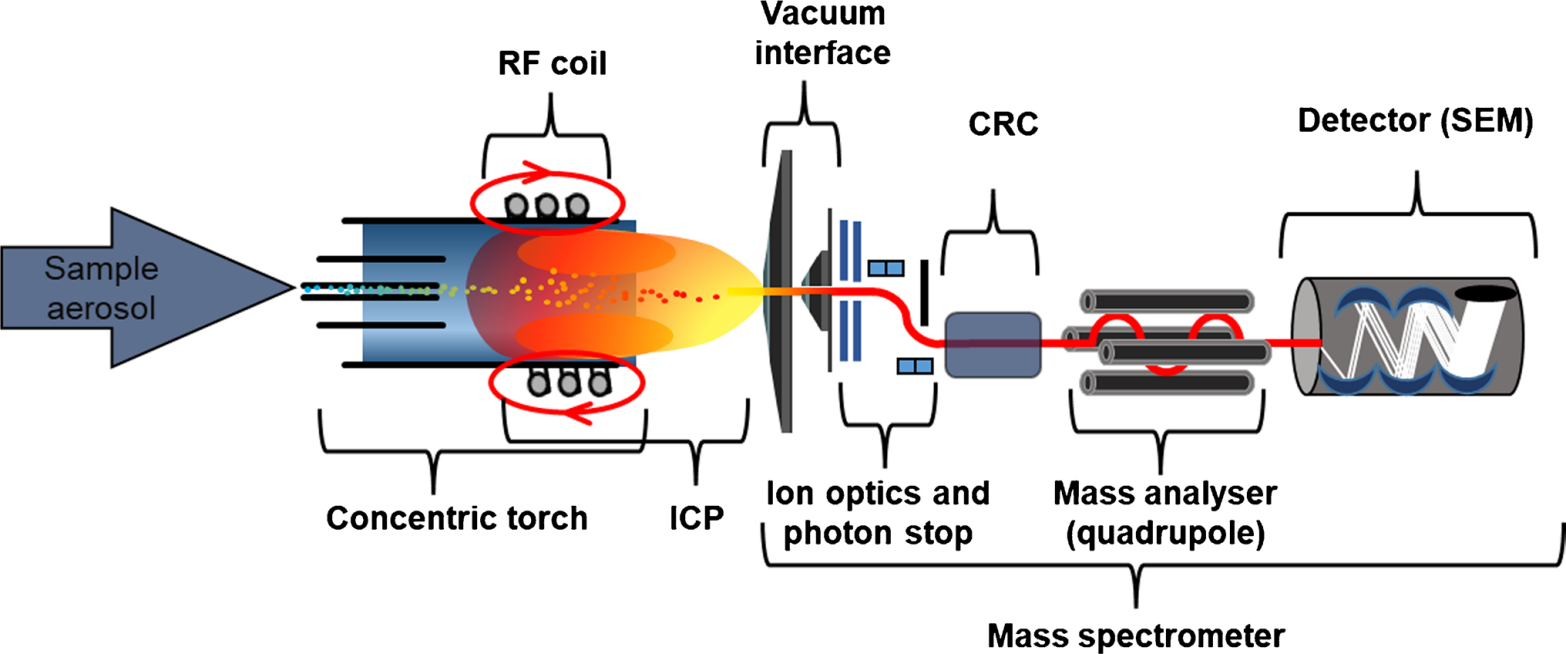
Schematic set-up of an ICP-MS system operating a collision/reaction cell (CRC) and a quadrupole mass analyser. A sample aerosol is introduced via liquid nebulisation or laser ablation, dried and atomised in the hot RF-powered ICP. Following ionisation, singly charged element cations are extracted from the plasma through a two-staged vacuum interface, focussed and guided around an attenuator (photon stop) to eliminate neutral particles and light. The operation of the CRC is optional and enables attenuation of spectral interferences, before targeted ions are separated according to their *m/z* and detected with a secondary electron multiplier (SEM)

Following the development of ICP as source to stimulate optic emission in the 70s [1, 2], ICP-MS was developed and commercially introduced in 1983 [3]. ICP-MS employs magnetic fields at 27 or 40 MHz sustaining a hot Ar-based plasma which reaches temperatures of up to 10,000 K [4]. At these temperatures, any molecular compound is immediately atomised, element cations are generated and consequently extracted for mass spectrometry. The ionisation degree of elements is mainly dependent on the plasma temperature and their first ionisation potential and can be calculated using the Saha equation. However, most elements of the periodic table are efficiently ionised to degrees exceeding 90% [5]. To sustain and contain the plasma, a high Ar supply is required consuming almost 20 L min^−1^. Different low-flow torch variants and alternative gases have been investigated but have not been adopted for commercial instrumentation [6–9]. Therefore, ICP-MS is associated with relatively high running costs. The processes occurring in the plasma produce relatively high ion yields which translate into high sensitivity. The underlying atomisation and ionisation mechanisms are generally not influenced by the chemical environment of elements and provide consistent sensitivity across different chemical species. On the one hand, this enables pragmatic solutions for absolute quantification of trace elements via species-unspecific (ionic) element standards [10]. On the other hand, the annihilation of any molecular information complicates the tracing of the molecular history and requires additional steps to obtain species-specific data. Generated ions are extracted in a coherent ion beam, which is transferred through a multi-staged interface into a high vacuum region [11]. Here, the ion beam is guided by ion optics for focusing and elimination of neutral particles (neutral Ar and light) before passing through a mass analyser to isolate specific isotopes according to their mass to charge ratios (*m/z*) and for subsequent detection (Fig. 1) [12, 13].

Different geometries and technologies are available in ICP-MS and especially the implementation of different mass analysers offers distinct advantages for certain analytic scenarios by reducing interferences, enabling rapid scanning and/or simultaneous analysis or providing increased ion transmission. The quadrupole mass filter was the first analyser introduced for ICP-MS and can still be seen as the work horse accounting for most applications and citations in routine and research [13]. Two prevalent reasons for this are its ease of use as well as its affordability.

In conjunction with a collision/reaction cell (CRC), ICP-QMS may target elements which are often interfered including transition metals (e.g. Mn, Fe), metalloids (As) and non-metals (e.g. S, P, Se) [14, 15]. CRCs offer different acquisition modes which enable attenuation of spectral interferences by exploitation of physical and chemical differences between the targeted isotope and the interference. Relevant strategies include here kinetic energy discrimination (KED) [16] to eliminate polyatomics and the exploitation of specific chemical reactions. For the latter, different chemical affinities can be harnessed by employing distinct gases in oxidations, reductions, charge transfer processes as well as adduct formations [14, 17]. Further information on the development and applications of the CRC can be found elsewhere [16, 18].

The quadrupole mass analyser consists of four parallel rods which are supplied with a combination of direct current (DC) and radio frequency (RF) voltages. The exact combination of both stipulates the bandpass and the mass resolution [19]. Analysing different *m/z* requires consequently a modulation of the DC and RF voltages, which makes the quadrupole a sequentially operating mass analyser. Figures of merit of many elements in ICP-QMS were significantly improved by the introduction of tandem mass spectrometry (ICP-MS/MS), which made the analysis of biologically important elements such as P or S viable. ICP-MS/MS was introduced in 2012 and incorporates an additional scanning quadrupole before the CRC enhancing abundance sensitivity and consequently the trace analysis of interfered elements by restricting CRC access to only one selected *m/z*. Controlling chemical reactions occurring in the CRC mitigates unwanted isobaric product ions, non-spectral interferences and matrix effects and improves CRC efficiency. These factors promote product ion scans, precursor ion scans and neutral mass gain/loss scans and overall contribute to improved limits of detection. The modes of operations and applications of ICP-MS/MS for trace elemental analysis in stand-alone configuration as well as in hyphenated techniques were reviewed by Bolea-Fernandez et al. [20] and Balcaen et al. [21].

The capabilities of quadrupoles are limited when it comes to mass resolution, fast multi-elemental acquisition, precision and ion transmission, which can potentially be a pitfall for specific biomedical applications. Sector field (SF)– [22–25] and time of flight (ToF)–based mass analysers [26–28] are increasingly integrated within the biomedical and life sciences. Especially, the latter analyser offers several advantages over quadrupoles which translate into unique opportunities regarding elemental bioimaging and single-event analysis, which will be discussed with more detail in the second part of this review. SF-based mass spectrometers were first developed for the high-resolution analysis of molecular compounds and were commercialised for ICP-MS in the 90s [29]. Most commonly, double-focussing SF instruments operating an electrostatic analyser (ESA) and a magnetic sector are employed to offer tuneable mass resolution (typically 300 in low resolution, 4000 in medium resolution and 10,000 in high resolution (m/Δm at 10% signal height)) and/or higher ion transmission to bypass relevant (polyatomic) spectral interferences and to improve limits of analysis. The ESA and magnetic sector are set-up in distinct orders (e.g. Nier Johnson and reverse Nier Johnson) and geometries (e.g. Mattauch-Herzog configuration). Especially, the Mattauch-Herzog configuration in which the magnetic sector is set up in a specific angle after the ESA and operated at a fixed magnetic field strength is relevant for the analysis of isotope ratios. In this set-up, different *m/z* follow laterally separated ion trajectories with different focal points. This offers the opportunity to employ multiple detectors (multi-collector (MC)) to acquire isotopes simultaneously and became relevant to determine precise isotope and element ratios for studies on fractionation effects caused for example by altered metabolisms and pathologies as addressed in a later section of this review. A drawback of a magnetic sector is a limited mass band pass. To acquire isotopes across a larger mass range, magnetic field strengths must be modulated which is much more time consuming compared against the scanning of quadrupoles or ESAs due to hysteresis effects. However, this may be bypassed by operation of a spatially resolving semiconductor detector which allows to acquire the entire mass range simultaneously (*m/z* 6–238) [30].

ToF technology for ICP-MS is becoming increasingly popular for the simultaneous analysis of isotopes across the entire mass range. Here, ions are collected from the ICP in packages and accelerated in an electrostatic field, separated in a field-free flight region and consecutively detected. During acceleration, all ions receive the same kinetic energy, and as such, high *m/z* travel slower than low *m/z*. The resulting flight time can subsequently be calibrated into a *m/z*. ToF technology for ICP-MS was first investigated in 1994 [31]; however, advantages could not be fully exploited due to various challenges which required further instrumental advances. Challenges were mainly related to the broad energy distributions of ions extracted from the ICP as noisy source, limited duty cycles, non-optimal flight paths and high abundant interferences saturating the detector (e.g. Ar^+^). Current instrumentation features notch filters and/or a CRC to eliminate highly abundant interferences and, compared to initial instruments, offers significantly improved sensitivity. However, compared against ICP-QMS, sensitivities are still considerably lower. A significant advantage of ICP-ToF–MS is the quasi-simultaneous acquisition of isotopes. Although the very mechanism of ToF is based on the time-resolved detection of ions travelling through the flight tube at different speeds, all ions are extracted from the plasma at the same point of time which increases precisions compared to ICP-QMS. Furthermore, ICP-ToF–MS offers increased mass resolution and acquires full mass spectra significantly faster than conventional ICP-QMS systems (acquisition speed is between 33 and 76.8 kHz) [26, 27, 32]. The fast multi-elemental acquisition results in large data sets which require dedicated software solutions, larger storage capabilities and high computational processing power.

## Stand-alone ICP-MS

### Biomonitoring

The capability to rapidly scan for several elements and isotopes across a vast linear dynamic range spanning over ten orders of magnitude made ICP-MS the most adequate approach to detect and quantify toxicologically relevant elements, to inquire occupational or environmental exposures and heavy metal poisoning [33]. However, many pathologies are caused or accompanied by a change in the concentration of endogenous elements, which opens interesting opportunities to interrogate elements as biomarkers [34, 35]. The capability of ICP-MS to determine the total levels of trace and major elements is becoming increasingly relevant in the medical realm for the determination of elements in biofluids and tissues to improve biomonitoring as well as to characterise pathologies based on element profiles and distributions.

However, it was the achievement of early atomic spectroscopy featuring flame photometry and AAS to elucidate the relevance of several elements in biochemical pathways [36, 37]. Along with other techniques including ion-selective electrodes (e.g. for Na, K), atomic fluorescence spectroscopy (e.g. for hydride forming elements As, Se) [38, 39] and flame photometry, AAS is still routinely applied for elemental analysis in the clinical environment (e.g. Li, Na, K, Ca, Mg) [37, 40, 41]. Essential elements are regulated within a narrow homeostasis and both deficiency as well as excess may lead to adverse physiological effects. However, non-essential elements may disrupt metabolic and signalling pathways as well if present at significant concentrations. The critical levels at which adverse effects are recognisable depend on a range of factors which include bioavailability, speciation and spatiotemporal distribution. For clinical applications, however, in-depth differentiation between metal species or element biodistributions is not performed and routine techniques therefore aim to determine the total element levels as a proxy for their toxicological impact. Knowledge of abundances of both essential and non-essential elements is extremely useful to recognise pathologies as well as to investigate natural and occupational exposures to potentially toxic elements. Exposures are often elucidated by analysing blood [42, 43], serum [44], plasma or urine [44–46]; however, also, other biomaterial such as saliva [47], hair and nails [48, 49] may be targeted. For the latter specimens, results are more prone to bias due to various factors such as age or habits and must be evaluated with great care [50].

Elements that are frequently analysed in the context of biomonitoring include Pb, Hg, Cd and As. The gradual improvement of AAS methodology including new atomisation techniques (delves cup, ET-AAS, CV-AAS), background compensation (e.g. Zeeman compensation) and new sources (e.g. continuum source AAS) [51, 52] enhanced capabilities and made the monitoring of multiple elements at trace levels viable. While AAS techniques offer fit-for-purpose analysis of elements in the clinical realm, ICP-MS is increasingly used to complement and substitute methods. The rather slow absorption of ICP-MS in the clinical landscape may be explained by its relatively high (running) costs, the requirement for more extensive expertise and the fact that previous techniques and infrastructure were sufficient to monitor elements within relevant levels. Nonetheless, the possibility to target variable and large panels of bio-indicative elements, improved detection limits and robustness, access to isotope data, high matrix compatibility and the versatility of ICP-MS to be applied for biomedical questions beyond monitoring are alluring and resulted in increasing interest and further implementation [37].

### Elements as pathological indicators

Most metabolic processes are reliant upon multiple trace and/or major elements and determining the endogenous element profiles may indicate pathological changes, which is insightful to characterise diseases caused or accompanied by a dysfunctional homeostasis [53]. The multi-elemental capability of ICP-MS is predestined to target larger isotope panels in the context of various pathologies and statistical analysis helped to identify significant up- and downregulations and to pinpoint elements with bio-indicative potential. For example, neurodegenerative pathologies including Alzheimer’s (AD), Parkinson’s (PD), Menkes, Wilson’s (WD) and Huntington’s disease as well as prion diseases, multiple sclerosis and amyotrophic lateral sclerosis (ALS) are known or suspected to be associated or accompanied with disrupted metabolisms of transition metals. This suggests the analysis of neurological tissue digests as well as body fluids for diagnostic or preventive purposes. Especially, the regulation and metabolism of the transition trace elements Mn, Fe, Cu and Zn were found to be relevant and were correlated with an increased occurrence, risk or progression of neurodegeneration [34, 54, 55]. Quantitative element data is not only valuable to identify pathologies, but also to inquire treatment options. For example, the role of Mn attracted increasing interest and was investigated in the pathogenesis of prion-based diseases and PD [56–58]. ICP-MS was used to calibrate the levels of Mn following chelation therapy and results suggested that this method may have a significant effect on the prolongation of survival in prion-based diseases [59]. However, besides Mn, many other essential and non-essential elements are relevant in the context of neurodegeneration to study pathogenesis, progression and treatment [34, 60–63].

As affected tissues are often only accessible post-mortem or following invasive biopsies, other more accessible specimens are often preferred to monitor for pathological indicators. In case of neurodegeneration, cerebrospinal fluid (CSF) is a suited material to study diseases of the central nervous system [64–68]. However, also urine [66], serum [67] and whole blood [68] are useful to pinpoint a disrupted homeostasis due to neurological pathologies. For example, González-Domínguez et al. [69] studied the metal profiles in serum during the progression of AD and mild cognitive impairment. The authors analysed a high molecular protein fraction and a low molecular metal species fraction from serum via ICP-MS and found that Fe, Cu, Zn and Al systematically change during continuing neurodegeneration. The low molecular species of Fe, Cu, Al and Co appeared to be involved in the pathogenesis of AD. Furthermore, hair was recently suggested as a proxy to evaluate the link between heavy metals (e.g. Hg and Pb) and cognitive impairment [70]. Hair is an interesting specimen which simplifies sourcing and may be adequate for population screening. For example, Tamburo et al. [71] studied the role of selected elements in relapsing–remitting multiple sclerosis. Levels of trace elements in the hair of patients were significantly different when compared to control samples which provided evidence for metabolic imbalance during pathogenesis. Further information on the application of ICP-MS in the context of neuropathology can be found in a review by Ha et al. [34].

It has been demonstrated that the altered metabolism in cancer cells induces noticeable changes in the levels of various elements which makes quantitative elemental analysis also interesting in the context of oncology. Lavilla et al. [72] studied the levels of elements in tumorous and adjacent non-tumorous tissues in paired biopsies from patients with colorectal cancer. Applying principal component analysis, distinct elemental profiles were found to play a role and linear discriminant analysis was subsequently capable to identify 90% of samples correctly by considering the elemental fingerprint of essential and non-essential elements. Wach et al. [73] investigated the diagnostic potential of major and trace elements in serum for bladder cancer patients. The elements Ca, Li, Ni and Sr were promising candidates for the early diagnosis [73]. Wozniak et al. [74] proposed to identify disrupted metal homeostasis caused by head and neck cancer by profiling elements in hair. ICP-MS was further found adequate to monitor the elemental regulations during cancer treatment. On the one hand, many chemotherapeutics rely on heavy metal complexes (e.g. Pt, Ru) and the uptake and accumulation in tumour cells can be determined directly, which was for example shown by Ghezzi et al. [75] in a breast cancer cell line. On the other hand, endogenous element profiles may also be interrogated to monitor treatment effects. Jiang et al. [76] determined elements during chemoradiotherapy of cervical cancer with the aim to identify biomarkers reflecting therapeutic effects. Following the administration of cisplatin and the application of radiotherapy, serum levels of Na, Mg, P, K, Ca, Se, Cu, Zn, Se, Sr and Ba dropped significantly, and ionic Al and Cu were correlated with treatment. Therapy-induced disrupted homeostasis may impact severity of side effects and elemental monitoring provides opportunities for counteraction.

Among other diseases, diabetes was investigated regarding elemental profiles. To investigate the role of 19 elements in type 2 diabetes, saliva and plasma was analysed by Marín-Martínez et al. [77]. Statistical data analysis revealed a correlation of distinct elements with chronic complications and metabolic control. Another study determined the abundance of 23 metals in newly diagnosed and untreated diabetic cases and controls. The results suggested that analysing element profiles may have potential to predict diabetes risk [78]. Further research on elements in diabetes involved research on the correlation of maternal, placental and cordonal metallomic fingerprints in gestational diabetes mellitus [79].

It is worth emphasising that besides the mentioned biofluids and tissues, virtually any biospecimen can be interrogated in ICP-MS regarding elemental profiles and a disrupted homeostasis. In the recent years, a range of studies started exploring rather untraditional specimens including faeces [80], tears [81], milk [82], breath [83], skin [84], bone marrow fluid [85], nasal exudate [86], follicular fluid [87] or seminal plasma [88] to just name a few examples. Especially microsampling of biological fluids seems to become more relevant to ensure minimal invasive procedures and to target scarce fluids. A recent review by Aranaz et al. [89] provides an overview of relevant techniques, specimens and their potential diagnostic values.

## Elemental speciation analysis in the medical sciences

### Speciation analysis—fundamentals

The function and biochemical impact of any element is depending on its species distribution. Due to the hard ionisation in ICP-MS, any molecular information is destroyed, and therefore, stand-alone ICP-MS is not capable to target individual species. However, the on-line hyphenation of separation techniques to ICP-MS enables the conservation of species information in the form of the retention or migration time. The coupling of liquid chromatography (LC) with ICP-MS was first described by Thompson and Houk [90] in 1986 and methods were soon diversified to target elemental species in the context of various scenarios. Today, a large variety of different separation techniques is available. Different LC methods provide complementary selectivity and have been applied in conjunction with ICP-MS to tackle biomedical questions. Relevant methods and mechanisms include normal phase and reversed-phase LC to enable separation of polar and non-polar species via adsorption and distribution mechanisms, respectively [91, 92]. Ion chromatography (IC) and hydrophilic interaction liquid chromatography (HILIC) provide separation of ionic and polar analytes using ion exchange, distribution and/or partitioning mechanisms [93, 94]. Size exclusion chromatography (SEC) is used to separate large biomolecules based on their molecular weight/size [95]. Furthermore, affinity chromatography can be employed to harness specific chemical interactions for the separation of biomolecules [96] (e.g. enzymes/ligands or antibody/antigen). However, stationary phases can also comprise of mixed modes, where specific separation mechanisms are applied simultaneously [97]. Separation of complex mixtures can further be achieved using two-dimensional LC, where eluting fractions are collected and injected onto a second column with complementary separation mechanism [98]. Gas chromatography (GC) provides separation of smaller, non-polar and volatile compounds [99]. Electromigration techniques enable separation based on electrophoretic mobility which can depend on size, charge and isoelectric point and mainly comprises of capillary electrophoresis (CE) and gel electrophoresis (e.g. SDS-PAGE) [100, 101]. While CE can be coupled on-line with ICP-MS, gels are usually analysed offline via ICP-MS or LA-ICP-MS. Also, techniques such as field flow fractionation for colloid analysis [102] as well as supercritical fluid chromatography (SFC) are becoming increasingly relevant for biomedical questions.

However, the hyphenation of separation techniques with ICP-MS often requires additional prerequisites and is not considered “plug and play”. For example, the coupling of GC with ICP-MS requires a heated transfer line and injector to avoid the condensation of compounds prior to their introduction into the plasma [99]; SFC-ICP-MS requires a backpressure regulator and splitter [103]; CE-ICP-MS requires a total consumption nebuliser, electrical grounding and a make-up flow; LC-ICP-MS methods using organic solvents require strategies to avoid the deposition of carbon as well as the deterioration of the interface and commonly oxygen is added and an inert Pt-interface installed. Basic considerations have been part of a series of fundamental and tutorial reviews, where readers new to this field will find further information [92, 104–106].

The most applied hyphenated technique for elemental speciation analysis is LC-ICP-MS, which offers a range of advantages and/or complementary information when compared to other speciation techniques such as LC–ESI–MS, MALDI-MS or spectroscopic techniques (e.g. IR, Raman). The broad range of stationary phases and consequently different retention mechanisms allow tailoring of methods to the species and matrix of interest. Furthermore, the species-independent sensitivity in ICP-MS allows the application of species-unspecific standards for absolute quantification. This is a major advantage when compared against molecule-selective techniques, for which ionisation efficiencies are depending on species and matrix. Both parallel analysis of element standards for external calibration and simultaneously acquired internal standards are applicable for absolute quantification. Especially isotope dilution analysis (IDA) is a technique with high utility for accurate and precise quantification of polyisotopic elements. While initially developed for stand-alone ICP-MS, it can be modified in a post-column approach to enable on-line calibration of transient signals [10]. More information on IDA is given in the context of isotope ratios in a subsequent section. Species-unspecific quantification in LC-ICP-MS has a high utility to quantify unknown or unstable species for which standards are not available. Additionally, the element-selective analysis in LC-ICP-MS reduces the complexity of samples by attenuating compounds which do not carry the targeted isotope. This is relevant for the analysis of biological matrices with extreme variations of chemical entities.

### Speciation analysis and elemental exposure

**Fig. 2:**
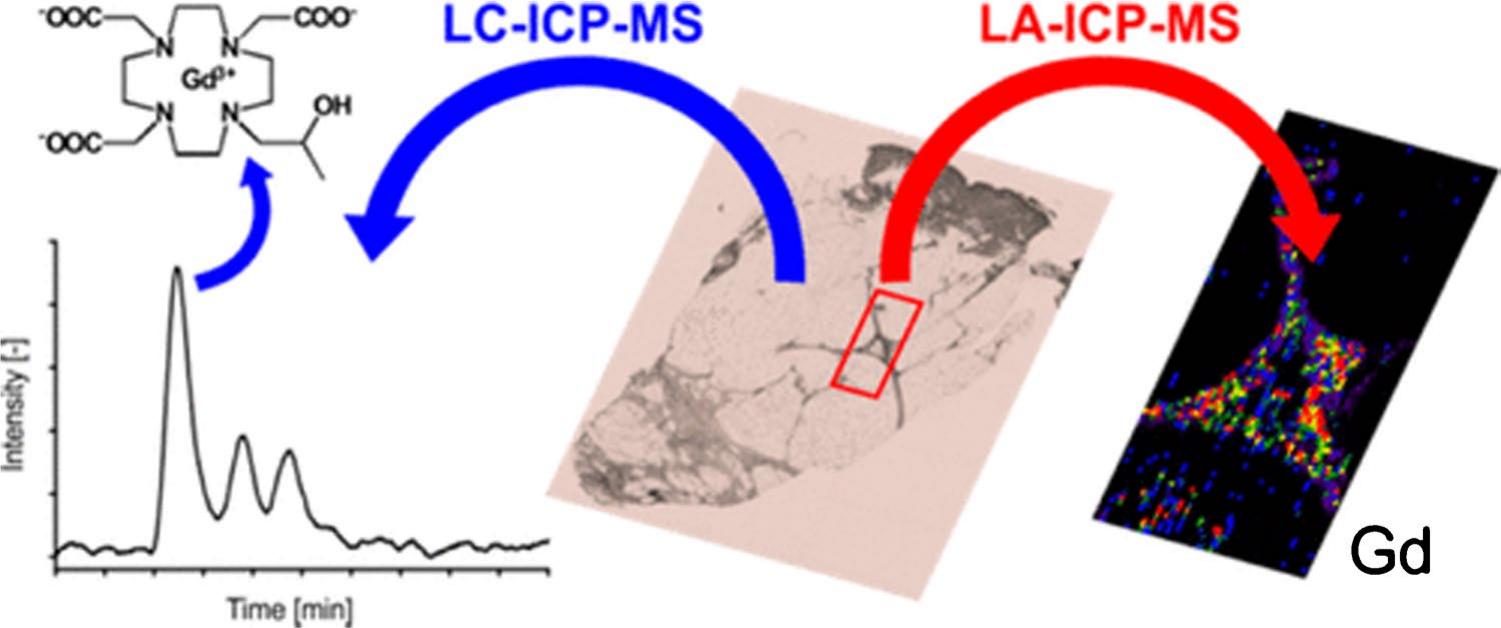
A skin sample of a nephrogenic systemic fibrosis patient, who underwent MRI examination years earlier, was extracted for HILIC-ICP-MS analysis. Complementary elemental imaging was used to locate the Gd in the tissue. Reprinted with permission from M. Birka, K.S. Wentker, E. Lusmöller, B. Arheilger, C.A. Wehe, M. Sperling, R. Stadler, U. Karst, Diagnosis of nephrogenic systemic fibrosis by means of elemental bioimaging and speciation analysis, Analytical Chemistry 2015, 87, 6, 3321-3328 [93]. Copyright (2015), American Chemical Society.

Elemental speciation analysis is relevant to study abundance and fate of natural and anthropogenic/toxic compounds in environmental and biological systems. While both systems may seem to have only little overlap on first sight, methodologies, targeted species and fundamental considerations are quite similar given that in both cases, matrices are complex and targeted species are usually present at trace levels. Furthermore, environmentally relevant species are often defined as such because they may have an adverse effect on physiology. Hence, the same elements and species are often targeted in both medical and environmental sciences and methods are often interchangeable [107]. One prominent example is the analysis of Gd-based contrast agents: contrasts in MRI imaging can be enhanced by the administration of polarising (high spin) Gd complexes. The retention of Gd species due to insufficient elimination may trigger pathologies and it was shown that Gd can be retained in several organs [93, 108] (Fig. 2). HILIC-ICP-MS was capable to find intact contrast agents in tissue extracts of patients who underwent Gd-enhanced MRI years before, as illustrated in Fig. 2 [93]. However, relatively high Gd dosages cause also a significant environmental discharge and LC-ICP-MS methodologies were applicable to find intact contrast agents in wastewater, seawater, riverine and drinking water [107, 110–112].

There is a large range of compounds and elements which were targeted since the inception of chromatography-hyphenated ICP-MS to study species-specific parameters including toxicity, bioavailability, translocation, bioaccumulation, biomagnification, degradation/transformation, fate and physiological impact [113–115]. In this framework, potential exposure routes are relevant and speciation analysis in diverse environments (e.g. air, wastewater, drinking water) and food stuff (e.g. plants, fish) as well as pharmaceuticals (e.g. therapeutics) have important medical implications. Several methods were developed to interrogate various species of elements including species of Fe, Cd, Cr, Te, As, Sn, Pb, I, Hg, Gd and Se in environmental and biological systems [107, 116–122]. Especially, As, Se and Hg have been studied extensively regarding metabolic pathways [115, 123–126], therapeutic contexts [126–129], pathologies or health impact [130–132]. Further information on clinical applications of LC-ICP-MS for element speciation can be found in a review by Delafiori et al. [133].

**Fig. 3:**
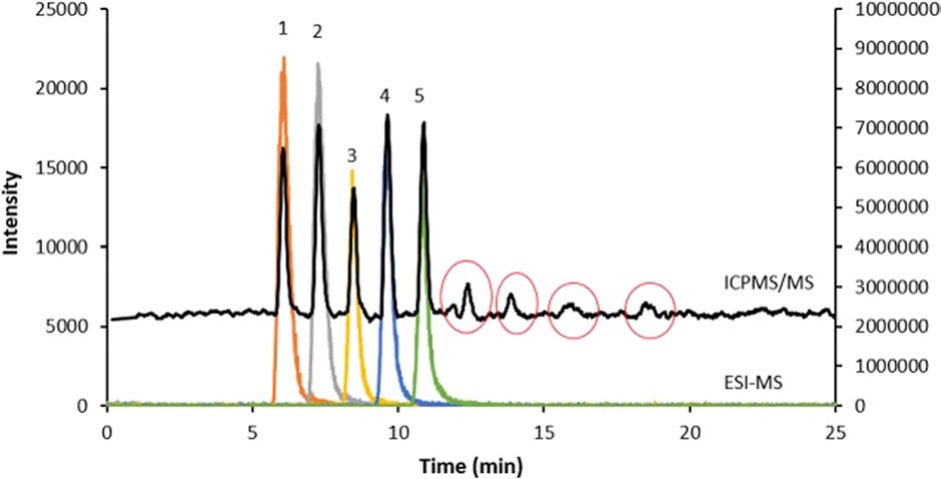
Overlay of LC–ESI–MS and LC-ICP-MS chromatograms of F-containing species. The LC-ICP-MS speciation methods enabled non-target F speciation analysis. Besides species identified via LC–ESI–MS, LC-ICP-MS detected additional species which would have been missed otherwise. Reprinted from Analytica Chimica Acta, 1053, N.L.A. Jamari, J.F. Dohmann, A. Raab, E.M. Krupp. J. Feldmann, Novel non-targeted analysis of perfluorinated compounds using fluorine-specific detection regardless of their ionisability (HPLC-ICPMS/MS-ESI-MS), 22-31 [109]. Copyright (2018), with permission from Elsevier.

While the utility of chromatography and migration techniques coupled to ICP-MS is obvious for the analysis of low levels of metal and metalloid species in complex matrices, technological advances suggested a whole new panel of elements to speciate. Especially, the introduction of ICP-MS/MS opened new avenues to target biologically/environmentally relevant non-metal entities containing for example halogens, S or P atoms [109, 134–137]. The analysis of halogenated compounds is interesting regarding biomedical applications due to their frequent and increasing incorporation in diagnostic or therapeutic pharmaceuticals [138]. As such, LC-ICP-MS/MS and -HRMS may study metabolites, distribution and fate of new non-metal-based drugs as well as exposures to a large panel of environmentally relevant entities including organic pollutants (e.g. pesticides, herbicides, polyfluorinated and -brominated substances) via their respective heteroatoms (P, S, Br, Cl, F) [99, 134, 139, 140]. Among the halogens, F is the most challenging element to analyse. Its high first ionisation potential prevents the formation of sufficient F^+^ for ICP-MS analysis and therefore complicates trace analysis [141]. Strategies to form metal-F ions (e.g. with Ba as metal) have been suggested and improve detection limits [109, 142]. Figure 3 showcases the possibility to employ LC-ICP-MS complementary to LC–ESI–MS to detect and quantify known and unknown (marked in red) F-based compounds [109].

### Analysis of proteins

As the analysis of non-metals was improved substantially, proteins with S-containing amino acids (cysteine and methionine) [95], nucleic acids [143], phospholipids [144], phosphorylated proteins [145, 146] and other biomolecules became detectable in ICP-MS. However, a significant portion of all proteins is also either associated with a metal co-factor or rely entirely on a metal-based catalytic active centre for functioning [147]. The possibility to reduce information density by targeting proteins containing only one specific isotope facilitates the investigation of the metal metabolism and promotes LC-ICP-MS for the investigation of metalloproteins. The biochemical regulation of respective metals is controlled in a narrow concentration range known as homeostasis, which can be seen as fine line between essential and adverse effects. Consequently, deficiency and excess of metals may trigger certain pathologies and hyphenated ICP-MS may target specific metals and proteins as bioindicators. However, accurate analysis requires considerations about the affinity between metals and proteins. The stability of metal-protein complexes is described in the Irving-Williams series and loosely bound metals (e.g. Mg^2+^, Mn^2+^) may detach from the protein framework during sample preparation and separation [92]. Furthermore, the isolation of protein fractions is often difficult and requires homogenisation and extraction steps which may compromise protein integrity. Therefore, it is noteworthy that elemental speciation analysis is often incompatible with classic biochemical and proteomic methods and its complementary application is with limitations. For example, the application of chemicals with high background levels of metals including gels and dyes but also harsh experimental conditions (e.g. high and low pH values, applications of high temperatures or electrical potentials) as well as certain extractants (organic solvents) and surfactants (SDS) may lead to contamination, denaturation or elimination of the metal factor. Therefore, techniques employing softer experimental conditions such as size exclusion chromatography or CE are often preferable [92, 95, 148, 149].

### Element speciation in pathologies

Elemental speciation has been employed to better understand metabolic pathways and to interrogate the levels of species and their correlation to various pathologies including diabetes [96] or stroke [150, 151], as well as in the context of cancer. A characteristic of cancer cells is their aberrant metabolism, which results in the accumulation of certain element species [152, 153], which can be targeted by LC-ICP-MS. However, the fact that many anticancer drugs are based on heavy metals/metalloids suggests the application of hyphenated ICP-MS to follow the metabolic routes of cancer drugs and to study their interaction with healthy and cancer cells. For example, LC-ICP-MS was employed to study Pt- [154], Ru- [100, 155], Os- [156], As- [129] and Ga-based anticancer agents [157] and provided a unique view on the generation of metabolites which are directly detectable and implicated with certain side effects and pharmacological parameters [155, 158, 159].

Substantial research has been conducted to identify and understand the impact of element species in neurodegeneration. Targeted and non-target approaches provided invaluable insights by correlating specific forms of degeneration with distinct element species containing Fe, Cu, Zn, Mn, Se, As, Hg and Al as detailed in a review by Michalke et al. [160] in 2018. In their review, authors concluded that the potential of speciation analysis is still not fully recognised in the field of neuroscience and suggested closer cooperation between neurologists/neuroscientists and analytical chemists for complementary research endeavours. Especially, in cases were biologists and physicians employ outdated techniques, LC-ICP-MS may provide entirely new perspectives. Previous studies demonstrated the potential of LC-ICP-MS in the context of for example AD, PD and WD and provided new insights regarding cognitive impairment and neurotoxicity [148, 161–168]. Studies have for example identified certain Se species as crucial for preserving brain function and preventing age-related degenerative disorders. However, an insufficient supply of Se may have a detrimental effect on brain cells by exacerbating neuronal dysfunction [169]. While some organic Se species are known to act as neuroprotectors, neurodegeneration is likely triggered from elevated inorganic Se species, stressing the need for speciation studies when assessing Se neurotoxicity. Vinceti et al. [166] evaluated the concentration of Se species in CSF samples from patients with mild cognitive impairment of non-vascular origin and whether conversion to Alzheimer’s dementia was triggered by them. Their results indicated that selenate levels in the central nervous system compartment may predict possible AD risks. Besides Se, other elements may be targeted to increase the understanding in neuropathology. One relevant element is Fe, which usually occurs at two different redox states (Fe(II)/Fe(III)), which show distinct redox chemistries that are closely related to the generation of oxidative stress and lipid peroxidation in the brain. In a study by Michalke et al. [170], a CE-ICP-MS method was developed to separate the two redox species and may be employed for the analysis of diluted cell lysates or CSF. In a study on SEC–ICP–MS, the authors further analysed Fe, Zn, Cu and Mn to gain a more profound understanding of relative abundances in size-resolved fractions from 24 paired human serum and CSF samples [171]. The study concluded that transition metals are strictly controlled at neural barriers of neurologic healthy patients. Altogether, it can be anticipated, that hyphenated ICP-MS will further expand the knowledge on neurodegeneration and improve our knowledge in prevalent diseases like PD and AD but also in age-related cognitive decline as well as to understand neurotoxicity as the result to the exposure to heavy elements.

## Elemental bioimaging

### LA-ICP-MS fundamentals

Understanding the role of individual elements in the biological environment requires not only knowledge on the element’s speciation but also its spatiotemporal distribution. Biological tissues comprise of highly compartmentalised micro- and nanometre-scaled structures containing countless anatomical features with diverse biochemical entities. Obtaining information on discrete locations of both essential and toxicologically relevant elements is therefore critical to estimate the biological role and impact. Visualising the up- and downregulation of elements is useful to recognise and locate the pathogenesis of various diseases, to develop strategies for intervention and to improve the understanding of biochemical pathways as well as metabolic disruptions. These motives have driven elemental bioimaging and inspired the technological framework of LA-ICP-MS, which was expanded by the application of labelled antibodies for mass cytometric applications as reviewed in the second part of the review. LA-ICP-MS pairs the high spatial resolution of LA with the high sensitivity of ICP-MS and is capable to map trace and major elements with concentrations ranging typically within the pg g^−1^ to the mg g^−1^ range at spatial resolutions between 1 and 100 µm (the higher the resolution, the lower sensitivity). Early applications employed LA-ICP-MS in the realm of geosciences; however, its potential to spatially resolve elements in complex biological structures was quickly recognised and developed. Since the first hyphenation of LA and ICP-MS in 1985 [172], the first bioimage [173], various technological milestones (e.g. ToF MS for ICP, fast wash-out cells) and dedicated methods (e.g. single-shot analysis and 3D imaging), today’s imaging systems are a platform technology which support highly sensitive, spatially resolved and rapid analysis of various elements while providing options for quantification, standardisation and multiplexed analyses [174]. Standardisation and calibration approaches require careful consideration of matrix-dependent ablation. The mass and size of the generated aerosol is strongly depending on the type of biological tissue and comparison of raw data as well as quantification approaches need to address these differences as explained in a subsequent section.

Pulsed excimer and solid-state lasers are most frequently applied in the field of elemental bioimaging and rastered in lines across tissues to gradually ablate and transport material for transient ICP-MS. Different software tools and dedicated standards can be applied subsequently to reconstruct and calibrate elemental distributions [175, 176]. The sensitivity and speed of analysis are depending on the laser spot size as well as the laser scan speed. The optimisation of these two parameters requires careful consideration about instrumental capabilities and a rough idea about the sample’s properties. The laser spot size stipulates the lateral resolution as well as the sensitivity. For example, decreasing the laser spot size by factor 3 reduces the ablated areas and consequently the absolute aerosol mass flow introduced into the plasma by factor 9 (3^2^) and therefore impacts sensitivity significantly. The maximum laser scan speed however depends on the pulse frequency and the aerosol wash-out time of the ablation cell as well as the scanning speed of the mass analyser. To obtain optimal figures of merit with scanning mass analysers, pulse frequency must be matched with the wash-out time and acquisition speed of the mass analyser to avoid aliasing and imaging artefacts [177, 178]. Highest speeds can be reached by employing cells with fast aerosol wash-out and novel designs achieve wash-out times in the low millisecond or even in the microsecond range [179]. The narrow signal width however limits the application of scanning mass analysers, which have dwell/scanning/settling times on a comparable time scale. Consequently, for scanning mass analysers, there is a maximum number of observable isotopes per laser pulse and pixel. The advance in ToF technology for ICP-MS improved LA-ICP-MS drastically and promoted biomedically important technologies such as mass cytometry imaging [180]. The possibility to detect all *m/z* (quasi) simultaneously enabled single-shot analyses, in which the aerosol of each laser shot and therefore each pixel is analysed for all *m/z*. Current improvements in ablation cell wash-out time promise to drive scan speeds as fast as hundreds to a thousand times of the dimension of the spot size. This immense scan speed results in the possibilities for rapid imaging, which is relevant for high sample throughput required in the clinical environment and further makes niche applications such as three-dimensional imaging more attractive. Fundamentals and applications of LA-ICP-MS for biological samples were recently reviewed by Doble et al. [174].

### Analysis of toxicologically relevant elements

LA-ICP-MS was employed to investigate the exposure to natural and anthropogenic compounds and was capable to follow the accumulation and translocation of diverse chemicals from various processes and environments. In cases where spatial resolution of elements is not required, LA-ICP-MS can be used as a solid microsampling technique to investigate for example dried body fluids like blood or urine [181]. Elegant options to reveal past exposure events also include the lateral analysis of hard tissues including bones, nails, teeth and hair. In some cases (e.g. nails and hairs), the time of exposure may be estimated via one-dimensional resolution of elements, which is for instance practical in forensic approaches [182]. However, in most cases, two-dimensional resolution of element distributions in biological samples is required to provide more detailed biochemical insights. As pointed out in the previous section, the behaviour, impact and distribution are not depending on the element itself, but its speciation. For example, hydrophobic species may accumulate in fatty tissues, overcome lipophilic barriers (e.g. the blood–brain-barrier, or cell membranes) via passive transport and follow different metabolic pathways. Polar species however may follow entirely different transport routes via active transport through ion transporters, for instance. To investigate the behaviour and distribution of distinct element species, LA-ICP-MS has been employed to study model organisms such as specific cell lines [28], *Drosophilia Melanogaster* [183], *C. elegans* [184] and *Daphnia magna* [185] in exposure experiments. However, for routine medical questions, exposure events are often vague, and little is known about the expected species. To establish a more holistic interpretation on the impact and meaning of elemental distributions, complementary molecule-selective imaging techniques are applicable and were reported in combination with LA-ICP-MS. Relevant techniques include here MALDI imaging, SIMS, DESI, LIBS or XRF/XANES [174].

### Bioimaging in pathologies

LA-ICP-MS has been employed to advance the physiological and biochemical understanding by targeting endogenous elements, therapeutics or diagnostic agents as well as natural and anthropogenic environmental contaminants. Several studies were published in the field of neurosciences where LA-ICP-MS proved to be invaluable to determine elements in microscaled, complex brain structures. Pathologies which were known for disrupted metal metabolisms were the logical choice for initial investigations of endogenous elements. The limited access to human biomaterial led to the development and frequent interrogation of different animal models as a proxy for human physiology and to improve the understanding of associations between pathologies and elemental distributions [174].

**Fig. 4:**
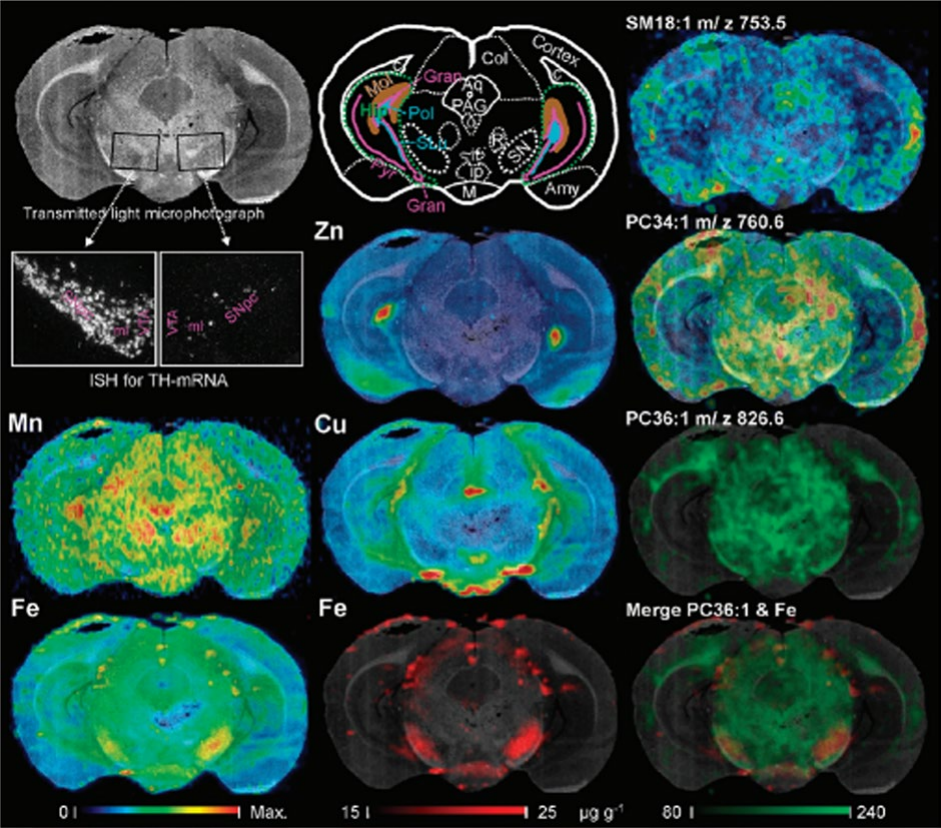
Multi-modal imaging approach integrating light microscopy underlain as background of each image facilitating morphological orientation, elemental concentration maps obtained by LA-ICPMS (left and middle column) and lipid maps obtained by MALDI-IM-MS imaging. Reprinted with permission from A. Matusch, L.S. Fenn, C. Depboylu, M. Klietz, S. Strohmer, J.A. McLean, J.S. Becker, Combined elemental and biomolecular mass spectrometry imaging for probing the inventory of tissue at a micrometer scale, Analytical Chemistry, 2012, 84, 7, 3170-3178 [186]. Copyright (2012), American Chemical Society.

The distribution of Fe was explored in brain matter in the context of PD and AD, and complementary techniques aided to provide more holistic interpretation between the relation of metals and biomolecules. While the initial causes of PD are not clearly defined, iron deposition has long been implicated with pathogenesis. Since the early work by Lhermitte et al. [187] in 1924, numerous studies have identified abnormalities in iron distribution in specific brain regions. LA-ICP-MS was employed to co-localise Fe and dopamine within the substantia nigra of mice to establish a risk index for parkinsonian neurodegeneration in the aging brain [188, 189]. Individual dopaminergic cells were determined by LA-ICP-MS and a relationship between dopamine and Fe was investigated in the midbrain. Matusch et al. [186] analysed 6-OHDA-lesioned rat brain sections using a combination of elemental and molecular mass spectrometry. Spatial distributions of Mn, Fe, Cu and Zn were obtained by LA-ICP-MS and correlated with the lipid distributions measured by MALDI-IM-MS imaging. Authors observed increased concentrations of Fe, Mn and Cu in the lesioned substantia nigra while monounsaturated lipid levels were decreased (Fig. 4). The same group demonstrated in a previous study that brain sections of mice subchronically intoxicated with 1-methyl-4-phenyl-1,2,3,6-tetrahydropyridin present alterations in midbrain levels of Fe and Cu [190].

**Fig. 5:**
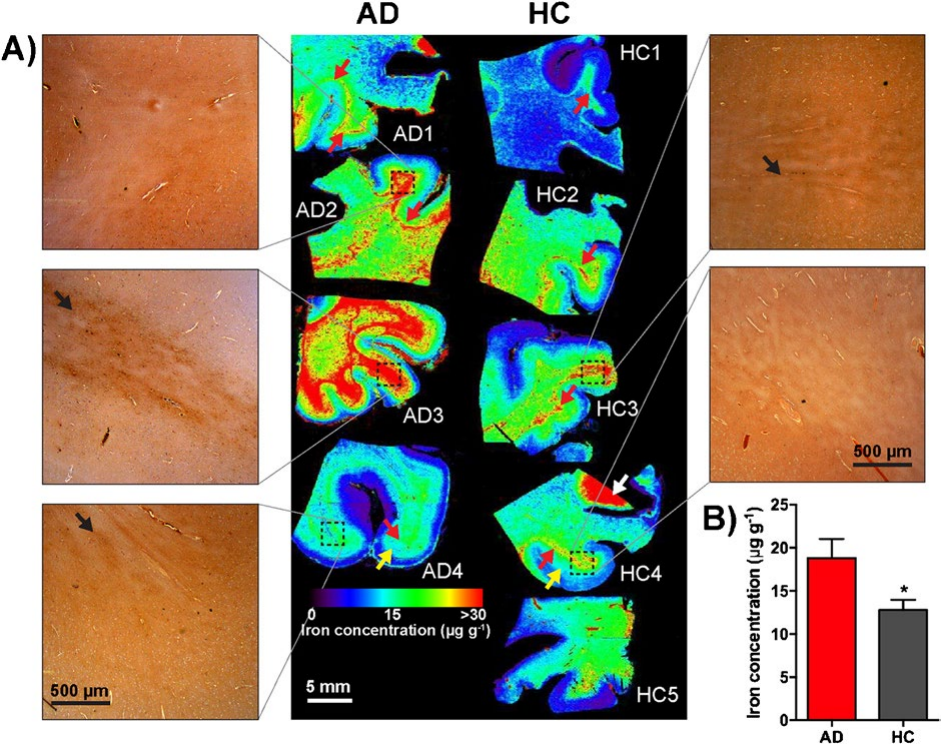
**A** Quantitative LA-ICP-MS imaging of iron levels in AD and a healthy frontal cortex and corresponding Perls images from selected regions of interest in all samples analysed **B** The combined white and gray matter iron levels in frontal cortex were significantly increased. Reprinted from NeuroImage, 137, D.J. Hare, E.P. Raven, B.R. Roberts, M. Bogeski, S.D. Portbury, C.A. McLean, C.L. Masters, J.R. Connor, A.I. Bush, P.J. Crouch, P.A. Doble, Laser ablation-inductively coupled plasma-mass spectrometry imaging of white and gray matter iron distribution in Alzheimer's disease frontal cortex, 128-131 [191]. Copyright (2016), with permission from Elsevier.

Similarly, impaired homeostasis of transition metals is believed to play a role in the pathogenesis of AD. Hutchinson et al. [193] conducted a study combining elemental and molecular imaging to investigate neurodegeneration. They successfully imaged the distribution of β-amyloid protein and trace metal ions in Alzheimer’s plaques. Cruz-Alonso et al. [194] performed simultaneous quantitative imaging of iron and ferroportin in the hippocampus of human brain tissues with AD and observed the trend that iron content increased in AD patients. Hare et al. [191] quantitatively assessed the iron content of white and gray matter in paraffin-embedded sections from the frontal cortex of AD and control subjects as shown in Fig. 5. Using the phosphorus image as a proxy for the white/gray matter boundary, they found that increased intrusion of iron into gray matter occurs in the diseased brain compared to controls. In a study by Rao et al. [148], the authors characterised the spatial and temporal brain metallomic profile in a mouse model of tauopathy, to provide insights into the potential interaction between tau pathology and iron. Using LA-ICP-MS in combination with SEC–ICP–MS, the study revealed an age-dependent changes in brain Fe and Cu levels in both WT and rTg(tauP301L)4510 mice.

**Fig. 6:**
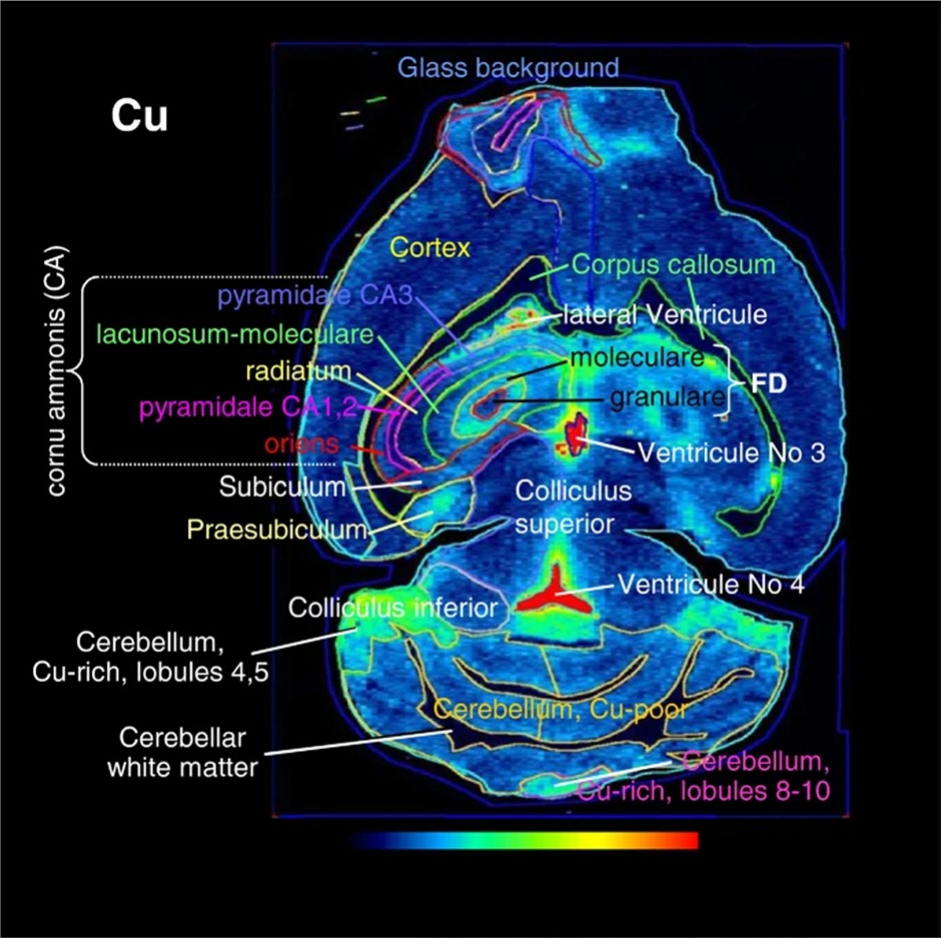
Different regions of the murine brain in which Cu content was measured by LA-ICP-MS and quantified. Reprinted from Springer Nature, BMC Neuroscience, 15, 98, 2014, Simultaneous monitoring of cerebral metal accumulation in an experimental model of Wilson's disease by laser ablation inductively coupled plasma mass spectrometry, S.G. Borau, U. Merle, R. Uerlings, A. Zimmermann, S. Weiskirchen, A. Matusch, W. Stremmel, R. Weiskirchen [192]. Copyright (2014), Boaru et al.; licensee BioMed Central Ltd.

WD is a rare, inherited autosomal recessive Cu overload disease, in which excess Cu accumulates in the liver, brain and other tissues. Therefore, imaging the spatial distributions of metals in tissue samples allows direct correlation of target regions and metal-associated processes. A study by Boaru et al. [192] reported an age-dependent accumulation of Cu, Fe and Zn in Atp7b-deficient mice as well as an elevation of these metals in human WD liver (Fig. 6). Hachmöller et al. [195] applied qualitative LA-ICP-MS for the investigation of human paraffin-embedded liver needle biopsy specimens. The analysed WD samples presented inhomogeneous Cu distribution and high Cu concentrations of up to 1200 µg g^−1^. Uerlings et al. [196] tested whether a AAV8-based therapy alleviates the cerebral Cu overload in the Atp7b null mice. Their results revealed that the delivery of AAV8-AAT-co-miATP7B can reduce the overall cerebral Cu content without affecting other metals.

LA-ICP-MS was further applied to resolve exo- and endogenous elements in cancer as bio-indicative markers to study tumorous tissues and treatment options. Several studies have exploited the capabilities of LA-ICP-MS as a sensitive imaging modality to visualise a variety of drugs (based on e.g. Pt [197, 198], Ru [199], Os [200]) in various organs affected by the cancer or associated with side effects including cochlea, liver, kidney, testis [201], bone [202], colon [203, 204] and ovary [203, 205] with the aim to investigate anticancer agent mechanisms. For example, elemental bioimaging has been applied to monitor drug penetration in tumour spheroids after incubation with chemotherapeutic agents (e.g. Pt-based drugs) [206, 207]. However, also, information on the up- and downregulation of endogenous elements may provide information about the cancer cells. The identification and analysis of essential elements may have a utility as bioindicator and may provide further insights into metabolic pathways. For example, the endogenous levels of Mn in tissues were investigated in the context of different cancers and their resistance towards radiotherapy [208]. It was concluded that Mn is a marker to predict the cancer’s response to radiation.

**Fig. 7:**
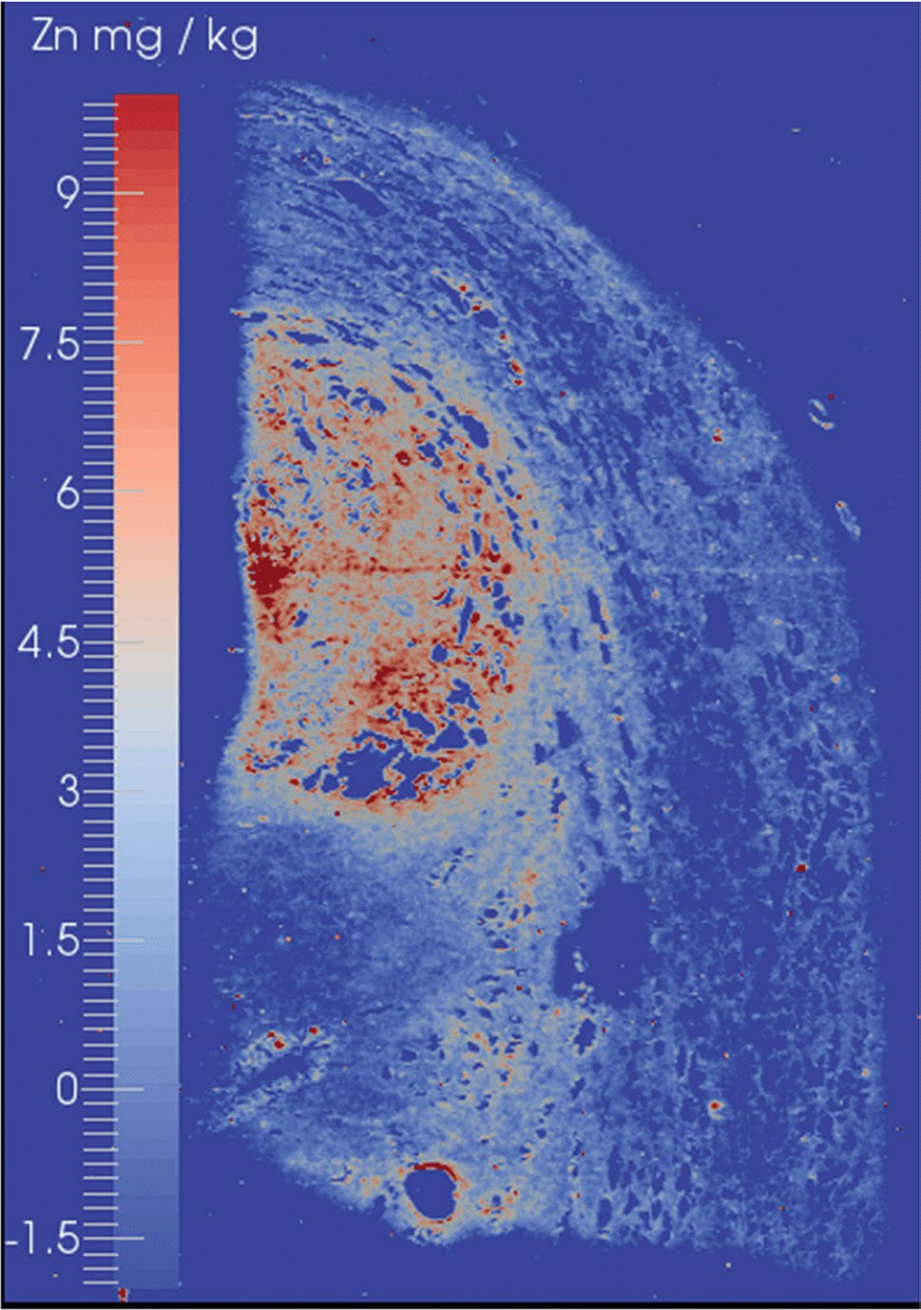
The quantitative Zn distribution was mapped in prostate cancer. The altered metal metabolism resulted into a Zn accumulation in the tumours area. Reproduced from Bishop et al. [210] with permission from the Royal Society of Chemistry.

The role of Zn has been investigated in several cancers (e.g. prostate [209, 210] and breast cancer [211]) and there is growing evidence that Zn-homeostasis is a keystone in health and implicated in various other diseases [212, 213]. Imbalance may contribute to cancer initiation and progression and LA-ICP-MS is well suited to reveal imbalances on the microscale. For example, Fig. 7 shows the Zn distribution in a human prostate cancer sample and the exaggerated Zn accumulation in the tumorous area is evident. Riesop et al. [214] suggested Zn as a potential biomarker of breast cancer as the histopathological malignancy grade can be directly correlated with Zn concentrations in invasive ductal carcinoma. Gonzalez de Vega et al. [215] examined matrix metalloproteinase 11 (MMP-11) by targeting its Zn co-factor as a proxy for its expression in breast cancer tissues and applied complementary MALDI-MSI to correlate Zn distributions with protein fragments following on-tissue digestion. The authors showed later that the protein MMP-11 may as well be targeted directly by incorporation of metal-coded antibodies, which will be considered with more details in the second part of the review [216, 217]. Other cancer types that were characterised by LA-ICP-MS included small-size induced tumours [218–220], glioblastoma [108, 221], neuroblastoma [222], melanoma [223], prostate cancer [209, 210, 224] and mesothelioma [225, 226], among others.

The application of laser spot sizes with dimensions on or below a cellular scale further suggests the application of LA-ICP-MS for the resolution of individual cells and small cellular structures. Although small spot sizes decrease sensitivity substantially, high-resolution LA-ICP-MS was previously demonstrated to be sufficiently sensitive to detect endogenous and therapeutic elements in single cells and cellular aggregates [227, 228] and suggested the interrogation of micro-scaled tumour models. This concerns for example tumour spheroids which can be analysed as small three-dimensional model to study parameters such as drug penetration in preclinical tests [206, 227]. However, single-cell analysis via LA-ICP-MS is becoming increasingly relevant in combination with labelled antibodies to probe the microenvironment of tumours and malignant cells as well as to identify and characterise rare cell subsets. This emerging facet of ICP-MS will be discussed in the second part of the review.

### Standardisation and quantification in elemental bioimaging

**Fig. 8:**
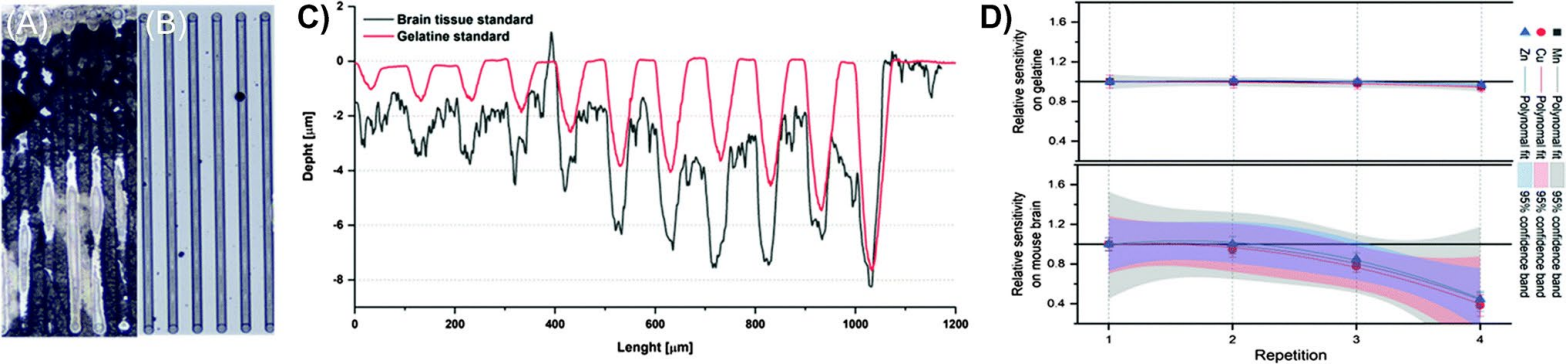
Mould-prepared gelatine standards are compared against matrix-matched brain tissue samples. Gelatine standards enhanced homogeneity, background levels, calibration range and control during ablation. **A** Homogenised brain tissue and **B** spiked gelatine standards are compared visually following LA-ICP-MS analysis. **C** compares the ablation depths and surface roughness of both standards. **D** compares repeated ablation of gelatine showcasing the enhanced control of material ablation. Reproduced from Westerhausen et al. [232] with permission from the Royal Society of Chemistry

One important and unique feature of LA-ICP-MS is its capability to quantify elemental distributions. While on-line quantification approaches have been presented and enable immediate calibration of each pixel as well as compensation of signal drifts during long run times [229–231], external calibration strategies are most commonly applied and involve the fabrication of matrix-matched tissue standards. Matrix matching of standards is required under the premise that the ablation process, the formation (e.g. particle size) and transport of the aerosol is depending on the biological matrix. Accordingly, analysing the same element at consistent levels in two different tissues may lead to significant differences. For accurate calibration, the tissue standards ideally comprise of the same matrix with defined elemental content. Homogenised animal tissues have frequently been used to model the complex biochemical and physical properties of human tissues [232, 233]. However, the vast diversity between different tissues/organs, species and even individuals makes the construction of ideal standards extremely challenging if not impossible. For example, the homogenisation step of standards intrinsically changes the tissue integrity which may have consequences for LA-ICP-MS. While the idea of matrix-matched standards for LA-ICP-MS is important for quantitative estimations and benchmarking, it is also relevant to recognise their limitations and pitfalls. On the one hand, standards are supposed to be reproducible, homogenous and precisely defined to enable laboratory- and instrument-independent quantification. On the other hand, matched matrixes should aim to simulate real biological tissues with extreme complexity regarding varying tissue domains, anatomical features and integrity. Any effort to model a perfect standard would end in circumstantial protocols which are hardly reproducible nor accurate. Consequently, it may be beneficial to find a compromise by identifying a standard material which matches biological tissues reasonably well, but is still easy to prepare, reproduce and trace [232]. While such material may not achieve the highest accuracy, it would allow comparable standards suitable for intra- and inter-laboratory comparisons and provide a high utility as a common benchmark. Especially in the biomedical sciences, the comparison to a control sample renders high accuracies less relevant and high reproducibility and traceability become more important to compare results across different cohorts, times-scales and laboratories [174]. A range of different materials has been presented in the past for matrix matching and consist of CRMs, modified CRMs, homogenised tissues [233], polymers [201] and gelatine [234]. The latter offers a range of advantages which translate into the possibility for more traceable and reproducible standards. Gelatine can be purchased at consistent and documented quality. It may be liquified to improve the handling and homogeneity during preparation steps and the actual LA experiment (Fig. 8). Furthermore, elemental background levels can be reduced using ion exchange resins which expands the calibration range and supports quantification of trace levels [232]. Finally, the physical dimensions (roughness and height) of standards can be controlled precisely by using for example specific moulds [232]. Consequently, gelatine-based standards may have the highest potential for spatially resolved (relative) quantification in biomedical approaches. Further information on calibration approaches, standardisation and basic considerations can be found in a range of reviews [174, 235, 236].

## Isotope ratio analysis

### Calibration via isotope ratios

Another facet of ICP-MS is its capability to determine precise isotope ratios. Highest precisions can be achieved with sector field–based ICP-MS, which is commercially available in different geometries. Especially, the Mattauch-Herzog geometry (multi-collector (MC)-ICP-MS) offers improved precisions reaching 0.001% by detecting isotopes laterally separated for simultaneous acquisition [237]. MC-ICP-MS is dominant in the environmental and geosciences to study fractionation effects, for provenance analysis or dating purposes, and its employment in the medical sciences is still a niche application.

Isotope ratios can be used for absolute quantification of polyisotopic elements in a technique named isotope dilution analysis (IDA). In view of complex matrices and sample preparation strategies, IDA provides advantages by offering precise internal quantification via a one-point calibration, which is independent from matrix effects and less prone to systematic errors during sample preparation. IDA is based on the addition of an isotopically enriched element standard. After homogenisation, any loss of analyte as well as any drift or fluctuation is mirrored for the enriched isotope and therefore compensated [10, 238]. IDA can be adopted for on-line quantification of transient signals produced in LC- and LA-ICP-MS [95, 229]. This offers robust analysis independent from instrumental drift or plasma fluctuations and allows instant calibration of unknown or unstable species. For on-line IDA, the enriched spike is added continuously to the sample flow and the spike’s isotope ratio is disturbed as soon as elemental species elute from the column or are delivered by the LA system [92, 229]. The transient change of ratios can be translated into concentrations and the interested reader will find more information in a tutorial review by Rodriguez-Gonzalez et al. [10].

### Tracer analysis

Isotope pattern deconvolution (IPD) is a technique that derived from IDA and features the application of element species with distinguishable isotopic abundances. It can be used to account for analyte gain/loss or inter-conversion, which is interesting in a biomedical context to elucidate the incorporation, translocation, species transformation and accumulation of elements due to physiological and pathological processes. IPD determines isotope ratios in a sample following the administration of enriched element species and deconvolutes the overall isotopic pattern to calculate the contribution from endogenous and the different external (experimental) sources. The technique was initially developed in 1997 by Hintelmann et al. [239] to study the artefactual formation of Hg species from inorganic Hg and was subsequently applied to understand the uptake and biotransformation during supplementation and fortification [240, 241], the impact of different chemical species [242], mineral metabolism [243], mechanisms in oxidative stress [244], in vivo implant degradation [245] and the impact of specific element species.

**Fig. 9:**
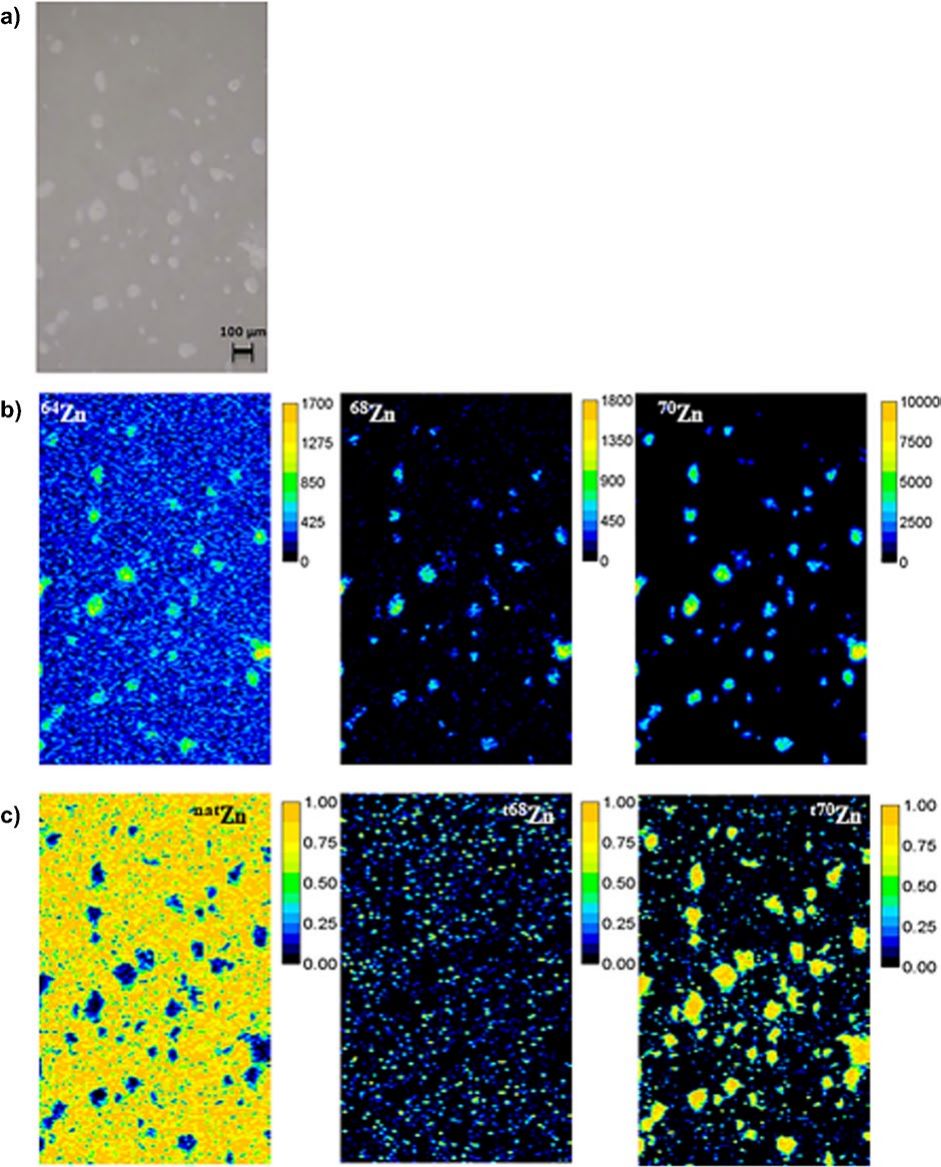
2D images of ^nat^Zn, ^t68^Zn (tracer) and ^t70^Zn (tracer) in HRPEsv cells, which were previously treated with Zn tracers. **a** Microscope image of the selected area in cultured HRPEsv cells, **b** qualitative images (in cps) of ^64^Zn, ^68^Zn and ^70^Zn, and **c** molar fraction images of ^nat^Zn, ^t68^Zn and ^t70^Zn. Reprinted with permission from S. Rodriguez-Menendez, B. Fernandez, H. Gonzalez-Iglesias, M. Garcia, L. Alvarez, J.I. Garcia Alonso, R. Pereiro, Isotopically enriched tracers and inductively coupled plasma mass spectrometry methodologies to study zinc supplementation in single-cell of retinal pigment epithelium in vitro, Analytical Chemistry 2019, 91, 7, 4488-4495 [249]. Copyright (2019), American Chemical Society.

Analysing stable isotope tracers via isotope ratios opens possibilities to study and understand trace metal metabolism without the need for radioactive tracers or labelling approaches. The high precision achievable via (MC)-ICP-MS is sufficient to analyse various isotopic tracers administered at biological concentrations. Especially, the spatially resolved determination of isotope ratios is interesting to observe the translocation and accumulation of tracers and suggests isotope ratio imaging via LA-ICP-MS. While biological applications were reported for plants and animals, medical applications are scarce [246]. Urgast et al. [247] demonstrated LA-ICP-MS as a microsampling device to follow two isotopically enriched Zn tracers at biological concentrations in rat tissues. Employment of MC instrumentation improved precision to spatially resolve isotope ratios [248]. In a recent study, Rodriguez-Menendez et al. [249] applied LA-ICP-MS to investigate Zn-based tracers and their uptake by HRPEsv cells. The authors used IPD to differentiate and map endogenous Zn and tracer isotopes, independently (Fig. 9). As noted by Urgast and Feldmann [246], isotope ratio analysis by LA-ICP-MS opens a wide range of possibilities for stable isotope tracer studies to investigate kinetics of trace elements or to understand physiology and pathologies.

### Isotope fractionation

Isotope fractionation occurs in various natural processes due to the more inert physical and chemical properties of heavier isotopes and results in small but measurable alterations in the isotope ratios. Especially, isotopes with large relative mass differences are affected in slow and low-energetic (bio)chemical reactions. This offers diagnostic opportunities, where altered isotope ratios may indicate an aberrant metabolism as a marker for exaggerated (e.g. cancer) or ceasing cell activity. Costas-Rodriguez et al. [222] studied the Cu metabolism and fractionation on a cellular level in the context of neurosciences using neuron-like cells and cells in a proliferating/tumour phase. The authors further evaluated the potential to employ the Cu isotope fractionation as a biomarker to diagnose liver cirrhosis [250]. In another study, Telouk et al. [251] investigated the potential of Cu isotope fractionation in serum as a diagnostic parameter for breast and colorectal cancer. Aranaz et al. [252] analysed various elements and the Cu isotope ratio in serum in a pilot study for age-related macular degeneration. Aramendia et al. [253] analysed Cu isotope ratios in serum for the diagnosis of WD and noted that the comparison of isotope ratios was more significant than comparing absolute concentrations. MC-ICP-MS was further applied to determine Mg isotopes ratios in serum of diabetes type 1 patients [254] and to analyse the Fe isotopic composition in patients with anaemia and chronic kidney disease [255]. Resano et al. [256] employed LA as a micro sampling device in conjunction with MC-ICP-MS to analyse Cu isotope ratios as a potential diagnostic marker for WD patients in dried urine and García-Poyo et al. [257] used a similar approach for the analysis of dried blood spots. LA-MC-ICP-MS may be applicable to visualise isotope fractionation, which was previously demonstrated to study provenance and migration of animals [258]. However, also diagnostic applications appear possible to pinpoint small tissue areas in the context of metabolism-induced isotope fractionation. Especially, on a cellular level or for small-scaled tumours, spatially resolved analysis may reveal local fractionation effects, which are decreasingly pronounced after homogenisation approaches.

## Conclusions and future perspectives

ICP-MS has distinct facets which offer strategies to analyse the role of elements regarding absolute concentrations, spatiotemporal and species distributions. The high sensitivity, isotope selectivity and vast dynamic range as well as technologies including the CRC and tandem mass spectrometry enabled detection of most elements of the periodic table making ICP-MS a viable technique for biomonitoring programs. This facet has a high clinical utility to reveal past environmental or occupational exposure events but further endorses the application of ICP-MS as a diagnostic tool to study element profiles in pathologies like neurodegeneration and cancer. It is predictable that ICP-MS will increasingly be employed in clinical research and routine to interrogate elements in various biomedical questions.

The scope of ICP-MS was significantly expanded by its on-line coupling to chromatography/electrophoresis and laser ablation. The possibilities to access species information and inquire distributions of elements enabled entirely new approaches to study the metal metabolism. Especially, in the context of pathologies like AD, PD and cancer, hyphenated ICP-MS promoted a better understanding of the disrupted metal homeostasis and inspired novel directions and discoveries. Furthermore, for the investigation of side effects and metabolic pathways of metallodrugs, ICP-MS techniques are becoming a logical choice. While some facets of ICP-MS are still niche applications like the analysis of isotope ratios, other facets and their potential have been recognised in the medical realm and are readily adopted. Especially, recent advances in elemental bioimaging and single-event analysis are currently implemented in the medical sciences and a clinical translation seems realistic as discussed in the second part of this review. These advances profit from the much-improved ToF analyser as well as immunochemistry methods and are applied in the context of mass cytometry, for example.

It is readily observable how hyphenated ICP-MS, associated techniques and novel methodologies/protocols are integrated into bioanalytical and biomedical workflows, tested in clinical trials and used to study novel therapeutics from complementary points of view. ICP-MS has a high utility in the medical sciences and its further integration into the clinical and research landscape likely depends on a transdisciplinary approach and cooperation between researchers of diverse disciplines.
